# Socio-cultural factors perceived to influence sexual behaviours of adolescents in Ethiopia

**DOI:** 10.4102/phcfm.v15i1.3865

**Published:** 2023-07-28

**Authors:** Semere G. Baraki, Gloria B. Thupayagale-Tshweneagae

**Affiliations:** 1 Department of Public Health, Menelik II Medical and Health Science College, Addis Ababa, Ethiopia; 2 Department of Health Studies, College of Human Science, University of South Africa, Pretoria, South Africa

**Keywords:** socio-cultural, influence, sexual behaviours, adolescents, Addis Ababa, Ethiopia

## Abstract

**Background:**

Adolescence is a period of transition from childhood to adulthood. It is the age of experimentation. They are vulnerable to the undesirable effect of sexual and reproductive health (SRH) problems such as human immunodeficiency virus, sexually transmitted infections and unsafe abortion and childbirth-related risks.

**Aim:**

To explore and describe perceived organisational, community and societal level factors that influence sexual behaviours among adolescents in Ethiopia.

**Setting:**

The study was conducted by public health care organisations, youth centres and non-governmental organisations in Addis Ababa, Ethiopia.

**Methods:**

A qualitative descriptive study design was conducted with purposively selected health professionals and adolescents in Addis Ababa from June 2019 to February 2020. The data were collected using in-depth interviews, key informant interviews and focus group discussions. Transcribed interviews were imported to ATLAS. ti 7 for coding, categorising and creating themes using thematic analysis. Lincoln and Guba’s model was used to ensure trustworthiness and ethical standards were applied.

**Results:**

Poor school involvement, social norms on sexual behaviour and lack of condom acceptability by the general population, financial problems and the gap in law enforcement were found perceived factors influencing sexual behaviour of adolescents.

**Conclusion:**

Adolescents are engaging in various risky sexual behaviours because of various organisational, community level and societal level factors, which emphasises the need to introduce social and culturally acceptable age-appropriate comprehensive sexuality education for adolescents and other multilevel interventions.

**Contribution:**

Provide an in-depth understanding of the influence of sociocultural issues related to adolescent sexual behaviour for health system stakeholders.

## Introduction

Adolescence is a period of transition from childhood to adulthood. It is the age of experimentation. Globally adolescents are vulnerable to the undesirable effect of sexual and reproductive health (SRH) problems such as human immunodeficiency virus (HIV),^1^ sexually transmitted infections (STIs)^2,3^ and unsafe abortion.^4^ Most young people enter puberty during their second decade of life.^5^ Because of a sense of independence and limited access to adequate and pertinent knowledge on SRH, they develop an interest in and engage in sexual and romantic relationships, and they also have a propensity to experiment with and engage in some harmful sexual behaviours^6^ and risky sexual behaviours can lead to many negative health related consequences such as STIs and unintended pregnancies.^7^

Sexually risky behaviour has a devastating impact on adolescent health, and it should be researched from different angles. Sexually transmitted infections, pregnancy and childbirth put adolescents at risk of unsafe abortion-related maternal morbidity and mortality and negatively impact their current and future lives regarding physical, social and economic problems.^8^

Studies done in Ethiopia indicate that there is a high prevalence of teenage pregnancies in which the pooled estimated prevalence of teenage pregnancy in Ethiopia was 23.59%.^9^ Although different strategies have been implemented to decrease risky sexual behaviours among adolescents, the problem of adolescent sexual risk behaviour persists.^10,11,12,13,14^ According to a study done in Ethiopia, preterm and low birth weight were found higher among adolescent girls than in adult women.^15^ Adolescents engaging in sexual intercourse before marriage, having multiple sexual partners, having sex without a condom and having sex under the influence of substances are some of the sexual practices reported among unmarried female youth in a semi-urban area of the Amhara Region, Ethiopia.^16^

We used social–ecological model as a theoretical framework for understanding the multifaceted and interactive effects of personal and environmental factors that determine behaviour. According to social–ecological model, a single factor cannot explain why some people or groups are more likely than others to engage in certain behaviours, because behaviour can be influenced by factors at several levels: the individual, relationship, community, organisational and societal levels.^17^ While the individual is responsible for engaging in a healthy lifestyle, the social environment (e.g. community norms, values, regulations and policies) greatly impacts individual behaviour. The social–ecological model recognises the relationships between the individual and his or her social and physical environment and that these relationships underlie health outcomes.^18^ This makes the model a suitable framework for inquiries of sexual behaviours among young people.

Norms and traditions are very important for the SRH of adolescents.^19^ For instance, sexual harassment is probably explained related to ideology and male dominance in society.^20^ According to a study done in Fiche North Shewa zone, Ethiopia indicated, youth with social connectedness such as family, school and religion were found with increased condom use, decreased number of sexual partners and reduced risky sexual behaviours. However, a high score of peer and social media connectedness was correlated with an increased number of sexual partners.^21^ Religion is an important factor, which can significantly influence the sexual behaviours of adolescents by delaying their sexual debut.^22^ The only way to guarantee the health care system’s capability, legislation, workforce, funding, infrastructure and data revolution for evidence-based decision, transparency and accountability is through government leadership, such as politicians, civil servants and lawmakers.^23^

Although there are many quantitative studies related to risky sexual behaviours in Ethiopia, there are limited qualitative studies that explore and explain why and how adolescents are engaging in risky sexual behaviours. This study aimed to explore perceived organisational, community and societal level factors that influence sexual behaviours among adolescents in Addis Ababa Ethiopia.

## Research methods and design

### Study area and period

The study was conducted in Addis Ababa, the capital city of Ethiopia. Addis Ababa has a mix of health facilities comprising 103 public health centres, 11 public hospitals, 33 private hospitals and 270 pharmacies.^24,25^ The data were collected from June 2019 to February 2020 in a purposively selected public hospital, public health centres, youth centres and one non-governmental organisation.

### Study design and study participants

This research used a qualitative descriptive approach. Qualitative descriptive research generates data that describe the ‘who, what, and where of events or experiences’ from a subjective perspective.^26^ From a philosophical perspective, this approach to research is best aligned with constructionism and critical theories that use interpretative and naturalistic methods. These philosophical perspectives represent the view that reality exists within various contexts that are dynamic and perceived differently depending on the subject; therefore, reality is multiple and subjective.^27^ The purpose of this study was to investigate the experiences, opinions and perceptions of adolescents and health professionals in adolescent youth clinics as health service providers on sexual behaviours and to explore the deriving factors influencing risky sexual behaviours. To achieve this purpose, adolescents and health professionals were included as the study participants using a purposive sampling technique.

### Sample size determination and data collection procedure

The researcher conducted eight key informant interviews with selected health professionals who were currently working in adolescents and youth health services, 12 individual in-depth interviews with adolescents who started penetrative sexual intercourse and five focus group discussions (three groups of males and two groups of females) separately. Data were generated through interviews, focus groups, memo writing and field notes through the entire involvement of the researcher with study participants or phenomena. Early preparation of an interview guide, appropriately worded questions and recording responses properly using memos and tape-recorder were some of the techniques used to increase the quality of information generated from the study participants. A preliminary literature review assisted the researcher with the questions used during data collection. The length of duration was 40 min – 75 min, 30 min – 75 min and 75 min –115 min for an in-depth interview, key informants’ interviews and focus group discussions, respectively. All the interviews were conducted at a convenient and preferred venue for the study participants.

The researcher spent much time in the field with study participants at the site to gain their trust and familiarise with them and the site of data collection prior to data collection. The researcher went back to participants in the study or community representatives in the area where the research had been working after transcription to verify data with them and get feedback on the accuracy of what was said and incorporate their feedback into the conclusion. In this research, data triangulation was applied using a variety of data sources including time, space and person. In addition, negative case analysis was applied to enhance, broaden, and validate the patterns emerging from the data analysis. Verbatim accounts were used to assure credibility. To maintain dependability, the researchers provided a detailed description of the process used and the researchers kept all the audio recordings, kept all the transcripts and kept all the subcategories, categories and themes. Besides, we justified how study participants were selected, the period of data collection and the methods of data analysis. To maintain conformability, the researchers remained faithful to academic and ethical requirements. The researchers kept all the transcriptions, categories, subcategories and audio-tape recordings in a safe place even after the publication of the research to ensure that research findings are the result of the experiences and ideas of the participants, rather than the characteristics and preferences of the researcher. In addition to this, the researchers presented detailed methodological approaches in this research. To maintain transferability, we purposely selected relevant study participants. During analysis, the researchers constantly compared, code with code, categories with categories, themes with themes and interviews with interviews from the beginning to the end of the data analysis.

### Data processing and analysis

We used thematic analysis because it is an appropriate method of analysis for seeking to understand experiences, thoughts or behaviours across a data set. The thematic analysis involves a six-step process: familiarising yourself with the data, generating initial codes, searching for themes, reviewing themes, defining and naming themes and producing the report.^28^ The principal investigator (S.G.B.) listened to the audio-recordings and transcribed them into the local language (Amharic). The principal investigator and two language experts independently translated the Amharic transcription into English yielding 375 pages of raw data. Transcribed data were translated into English by the principal investigator. Inductive coding was applied where themes were derived from the empirical evidence related to this study. Related codes were combined to form themes. Finally, in presenting the finding, participants’ quotes were used to elaborate on the umbrella theme being discussed. The analysis was done using ATLAs. ti 7 software application.

### Ethical considerations

Ethical approval was obtained from the University of South Africa, Department of Health Studies, higher degrees committee with the reference number Rec-012714-039 (NHERC) and from Addis Ababa city administration Public Health Research and Emergency Management Core Process (AACAPHREM) with the reference number of አ/አ/ጤ/30/703/227. Permission was requested from the heads of public health centres, hospital, Family Guidance Association of Ethiopia (FGAE) and youth centres. Participants were given all the relevant information relating to the study that includes the title, purpose of the study, benefits and any potential risks. Participants have had the opportunity to ask questions freely without any fear. The researcher emphasised that participation was voluntary and that the participants could withdraw from the research at any given time without any repercussions. The contact details of the researcher, supervisory teams and relevant ethics committees were provided to the participants on the consent and assent forms that were attached. The data were collected from anonymised responses; security techniques were maintained starting from data collection, analysis and presentation. All the data were protected using a password, and the audio was immediately copied to the computer and saved using a password and deleted from the tape recorder. Participants were assured of strict confidentiality regarding the information collected; that only aggregated data will be made available as part of scientific and public dissemination. Written informed consent was obtained from the individuals and/or minors’ legal guardian or next of kin for the publication of aggregated study results and any potentially identifiable data included in this article.

## Results

### Sociodemographic characteristics of study participants

The socio-demographic characteristics of each participant are described as presented in Table 1.^29^ Individual in-depth interviews were conducted with 12 sexually active adolescents. Adolescents who had experienced penetrative sex in their lifetime were included in the individual in-depth interview.

**TABLE 1:** Socio-demographic characteristics of adolescents participated in individual interviews in Addis Ababa, Ethiopia, 2021 (*n* = 12).

Participants	Age	Sex	Religion	Grade	Living situation	Ever had penetrative sex in lifetime	Youth centre sub-city
A1	19	Female	Orthodox	7	Living with partner	Yes	Lideta
A2	18	Male	Orthodox	12	Living with parents	Yes	Akaki Kality
A3	18	Male	Orthodox	12	Living with parents	Yes	Akaki Kality
A4	18	Male	Orthodox	10	Living with mother	Yes	Kirkose
A5	19	Female	Orthodox	10	Living with parents	Yes	Kirkose
A6	18	Male	Orthodox	12	Living with father	Yes	Arada sub-city
A7	19	Male	Orthodox	9	Living with mother	Yes	Arada sub-city
A8	18	Female	Orthodox	12	Living with partner	Yes	Kolfe keraniyo
A9	19	Female	Orthodox	12	Living with parents	Yes	Nifas silk lafto
A10	17	Female	Orthodox	10	Living with grandmother	Yes	Kirkose
A11	17	Female	Orthodox	9	Living with aunt	Yes	Kirkose
A12	18	Male	Orthodox	11	With both parent	Yes	Kirkose

*Source*: Baraki SG. Socio-cultural influences on adolescent risky sexual behaviour in Addis Ababa community: grounded theory approach [PhD Thesis]. Pretoria: University of South Africa; 2021

A focus group discussion (FGD) was conducted with adolescents. The maximum number of participants in one group was seven, and the minimum was six in the groups. The participants in FGD1, FGD2 and FGD4 were all males, while the participants in FGD 3 and FGD 5 were all females.

Key informant interviews were conducted among eight health professionals who had the experience of working with adolescents’ SRH. The socio-demographic characteristics of health professionals who participated in the study were also presented. The average age of the study participant in this group was 35 years. The oldest was 54 years old, and the youngest was 24 years old. They had an average of 13 years of work experience, with a maximum of 32, and a minimum of 4 years of work experience. There were four females and four males. Regarding their profession, six of the total health professional participants were nurses, while the rest were health officers. All participants are working in the health centre, hospital, non-governmental organisation (NGO) and youth centre, respectively. The term code in Table 2^29^ indicates the code of the study participants during data collection and data analysis, and the sub-city indicates the living areas of the study participants (Table 2).

**TABLE 2:** Socio-demographic characteristics of health professionals who participated in individual key informant interviews in Addis Ababa, Ethiopia, 2021 (*n* = 32).

Age	Sex	Profession	Educational level	Marital statues	Work experience	Sub city	Religion	Participants’ code
54	Male	Nurse	Degree	Married	32 years	Addis Ketema	Orthodox	HP1
27	Male	Nurse	Diploma	Single	5 years	Yeka	Protestant	HP2
24	Female	Nurse	Degree	Single	4 years	Bole	Protestant	HP3
26	Male	Nurse	Diploma	Single	4 years	Bole	Orthodox	HP4
32	Female	Nurse counsellor	Diploma	Single	7 years	Bole	Orthodox	HP5
29	Female	Nurse counsellor	Degree	Married	6 years	Gulel	Orthodox	HP6
54	Male	Health officer	Degree	Divorced	35 years	Lideta	Protestant	HP7
40	Female	Health officer	Degree	Married	15 years	Bole	Orthodox	HP8

*Source*: Baraki SG. Socio-cultural influences on adolescent risky sexual behaviour in Addis Ababa community: grounded theory approach [PhD Thesis]. Pretoria: University of South Africa; 2021

In this research finding, three themes, seven categories and 18 sub-categories were identified. Theme one is organisational, theme two community-level factors and theme three is societal-level factors. The themes, categories and subcategories are briefly summarised in Table 3.^29^

**TABLE 3:** Themes, categories and sub-categories of the analysis identified in the qualitative analysis of adolescents and health professionals in Addis Ababa, Ethiopia, 2021.

Themes	Categories	Sub-categories
Organisational-level factors	Healthcare factors	Limited resources in the youth centre
		Health professional’s gap in counselling and demonstration
		Fear attending public health care organisation
	School-level factors	Having romantic relationships in school
		Ease of access to substance
		Limitation of comprehensive sexuality education
	Business organisation factors	Expansion of provocative business organisations
Community-level factors	Social norms on sexual behaviours	Discouraging culture of early initiation of sex
		Discouraging culture in the community on having multiple sexual partners
		Religious belief regarding early initiation of sex
	Lack of condom acceptability by the general population	Negative community attitude towards condoms
		Fear and shame of buying and carrying a condom
Societal or macro level factor	Financial problem factors	Joblessness
		Sex for material or money gain
		Family poverty
		Rural-urban migration
	The gap in law enforcement	Poor law enforcement of age restriction for adolescents
		Selling drugs without prescription

*Source*: Baraki SG. Socio-cultural influences on adolescent risky sexual behaviour in Addis Ababa community: grounded theory approach [PhD Thesis]. Pretoria: University of South Africa; 2021

### Theme 1: Organisational-level factors

The sexual risk behaviour was used to explore the lifetime risky sexual activities of adolescents. We asked about the presence or absence of vaginal sexual intercourse, early sexual debut (before 18 years of age), having multiple sexual partners and inconsistent condom use in their sexual practices. Behaviours that contribute to unintended pregnancy and sexually transmitted diseases, including HIV, were considered risky sexual behaviours in this research. Healthcare organisations and school related such as poor school involvement factors are identified as the organisational-level factors that drive adolescents to engage in risky sexual behaviours.

#### Category 1.1: Healthcare-related factors

The narratives of study participants indicated that limited human and material resources, problems of attitude and knowledge, being older age of health professionals and unfriendly approach between health professionals and adolescents discourage adolescents from attending health care organisations. Health professionals working in youth centres listed a lack of diagnostic material for HIV and pregnancy diagnosis, penile models and sterilisers. A small number of trained professionals were also some of the human resources listed by the study participants. There was a gap among health professionals in both communication skills and condom demonstration with adolescents. Both adolescents and health professionals stated that there are some health professionals who are aggressive and have poor communication skills. These adolescents fail to tell them about their problems, and hence they do not get the appropriate treatment. In addition to this, health professionals do not know about condom demonstration; rather, they mainly focus on sexual abstinence. Few interviews also indicated that health professionals have a negative attitude towards adolescents who attend healthcare organisations and adolescents do not go to healthcare organisations because of the negative judgemental attitude of health professionals towards them.

‘For those who get the training, we have only one penile model. We call 50 youth for the training. Fifty of them want to touch it. Imagine showing one [*laughing*]. It has been almost two months since the voluntary counselling and testing service stopped. It is because we do not have test kits. STI diagnosis and treatment service is not provided here, it is not functional here, we refer them to the health centre.’ (HP3, age 24, female)

‘From my own experience, we adolescents do not go to the hospital. This is because they [*health professionals*] judge negatively and considered us rude. So, whatever happens, we try to solve it by ourselves.’ (A8, age 18, female)

#### Category 1.2: School-level factors

Based on the narratives of the study participants, the school environment was one of the common areas to have a sexual relationship despite inadequate education about SRH. Having sexual relationships with inadequate knowledge and skills on SRH influence adolescents to get involved in risky sexual behaviours. School is one of the most important places in which adolescents communicate and are influenced (pressured) to start sexual relationships and intimacy. In schools, they usually play different games that push them to sexual intercourse; they kiss each other and even engage in sexual intercourse in some schools inside toilets and unlocked classes. Substances are easily accessible in schools. Students use the substance in the school environment and inside the school as well and that leads to risky sexual behaviours. Students bring alcohol, weed and drugs like Domadol (Capsule) and use them in the school compound. There are shisha, khat houses and bars also available in the school compound; students miss classes to go to these houses and engage in substance including alcohol abuse leading them to engage in unplanned, unsafe sex. Comprehensive sex education is very limited in the school environment. Study participants listed reasons for this, for example, the biology teacher does not teach in detail about SRH; there are very weak co-curricular clubs, and there is limited curriculum integration:

‘If you go to unlocked free classrooms (school), you will find students playing a game in a group. You sit forming a circle… For example, if it is said “Mr X loves Miss Y, the order might be “Kiss her until we tell you to stop.” In the game, your sexual feelings are aroused.’ (A5, age 19, female)

‘Many nightclubs are open here. The nightclubs are near to the schools. The students see them when they go and come out of school. It influences the students.’ (FGD2, age 16, male)

‘Neither their parents nor the schools provide them with reproductive health education based on their age level. This is because reproductive health education is not well integrated with the curriculum.’ (HP2, age 27, male)

However, few participants indicated that health professionals are sometimes invited from outside and educate them once or twice a year:

‘They teach us many things. Twice a year, they give us training on substance use and HIV and AIDS by inviting health professionals. I think it is the health professionals that helped us protect ourselves.’ (A4, age 18, male)

#### Category 1.3: Business organisation factors

Growth in the number of business organisations such as brothels, traditional alcohol sellers, hashish, khat house and illegal (without a licence) houses was perceived as risky for adolescents. Professionals and adolescents listed well-known villages including Gerji, Saris, Kera, Bole, Chichinia and Gazebo as places for substance use. In most of these areas, there are sex workers; adolescents usually engaged in sexual intercourse with them. Some of those houses seem legal but inside they are giving different illegal services, as mentioned below:

‘There are khat houses, hashish houses, and liquor houses, which are called *Gazebo*. Many of the houses are given by the government [*poverty housing relief misused*]. They are old. For example, if you go to the village, which is called DC, for foreigners it has a different meaning, here in this village, I think you have heard about it, it is called Dirty City. The Dirty City means the place where many prostitutes live and the people who do not have money could rent a bed.’ (HP4, age 26, male)

‘The houses appear like legal places, one would think is a normal coffee house, But inside many illegal activities could be practised.’ (FGD2, age 17, male)

### Theme 2: Community level

Social norms on sexual behaviour and the lack of condom acceptability by the general population were categories identified in the study.

#### Category 2.1: Social norms on sexual behaviour

According to the narrative of the study participants, the community in urban Addis Ababa discourages or does not approve of early sexual intercourse with either one or multiple sexual partners. The Ethiopian culture and almost all religious organisations strictly advise abstinence from sex before marriage. Sexual intercourse before marriage is culturally not acceptable. In addition to this, almost all the participants explained that it is not acceptable by religion as well. This is discussed in the following two subcategories:

‘When you see it from the religious perspective, there is a religious-based wedding or holy matrimony. If you want to marry based on your Ethiopian Orthodox Church with a holy matrimony ceremony, you have to be abstaining from sexual intercourse. Therefore, it means that you should not do it [*sex*].’ (FGD3, age 19, female).

‘I do not think it is [*multiple sexual partners*] acceptable in the community. Some individuals do it, but they do not like it when others do it. You may face discrimination among the community.’ (A12, age 18, male)

But few study participants described that early initiation of sexual intercourse among females is strictly considered unacceptable when it is compared to male adolescents:

‘Second, if a woman said, “I have never experienced sex,” people would say that the woman is cultured and has good behaviour. But if a woman says that she has had sexual intercourse, she is considered cheap in the eyes of the man. He assumes that she sleeps with every man. So, I never speak of having sex because, in the man’s view, I will be seen as rude.’ (A5, age 19, female)

#### Category 2.2: Lack of condom acceptability by the general population

Although there were social taboos about the early initiation of sex, the general community had a negative attitude towards condoms. This type of negative attitude causes fear and shame in buying and carrying condoms among adolescents and finally drives adolescents to have unsafe sex during their exposure to sex. When adolescents need to buy and use condoms from a shop, pharmacy or public and private healthcare organisations, the people you find around them show a negative attitude towards such adolescents. Even when adolescents have (condoms) for safety, the community judges or assumes as adolescents started sexual intercourse. Adolescents fear discussing or buying condoms because they assume it is taboo in their culture. This issue was raised by both health professionals and adolescents. They claim that discussion about condoms used by their family members as either initiating sex or is a sign of being involved in sexual intercourse. If adolescents are found buying condoms by other community members or holding them, they may have an excuse that they bought for their friends. Society does not encourage adolescents to buy or use condoms:

‘The community does not accept condoms. These days, if the condom falls out of your pocket and somebody sees it, they will have a bad impression. They considered you as bad and indecent. You may be discriminated against and not allowed to meet your friends. Therefore, the community sees it as bad and it does not have acceptance.’ (FGD2, age 17, male)

‘Look at here. There is a condom on the wall. However, they do not take it now. They take it at lunchtime when the workers are out or they take it when the worker has gone home after 11:00 o’clock local time (17:00). They take it on Saturday and Sunday. When we are at the emergency, many youths come and take it.’ (HP7, age 54, male)

### Theme 3: Societal or macro-level factors

#### Category 3.1: Financial problem factors

Study participants reported three different financial concerns that drive them to risky sexual behaviours, namely joblessness, sex for money or material and family poverty. They expressed that if adolescents are jobless, they are inclined to look to other avenues for survival. Sex for the purpose of material and monetary gain is common among urban adolescents in Addis. According to the narratives of study participants, transactional sex with older people (‘sugar daddies’, ‘sugar mommas’) is experienced by both girls and boys, although female adolescents are more engaged in it than males. They get money and different gifts such as watches, perfumes, jewellery, mobile apparatus, shoes and laptops. The participants also stated that family poverty is also another financially related factor listed by study participants. Very few study participants explained that when the family of adolescents encounter a financial problem, they push their children to commit sexual intercourse for the sake of getting money. On the other hand, adolescents may sleep with their families because of a shortage of rooms and thereby can observe what their families are doing in relation to sexual intercourse, and this can lead them to experiment. In addition to this, some poor families use their homes for renting a bedroom, which leads to rape/sexual harassment for adolescents living there. According to the participants’ opinion, many youths come from the countryside with the hope of attaining work opportunities in the cities. However, when they come to urban areas, they face many challenges and some lead them to engage in risky sexual behaviours because it would be their first time of independence from parental control; they live alone and have no support system in the city:

‘I agree with participant three because most girls in government schools come from poor families. If I ask my parents to buy me a laptop, they cannot afford it. They earn little just enough to feed us and to buy clothes. So youngsters have sex to get what they want.’ (FGD5, age 17, female)

‘A mature person who very much respect tried to offer something of value to me in exchange for sex. He tried to convince me by saying “I will give you something that you lack”. When you don’t have money, they will say “I will give you money”.’ (A9, age 19, female)

‘This is clear. For example, if you are a bed renter and have a young boy or girl, the drunken person or the one who wants to have sex may come to that place to rent a bed. I think this thing can influence them [*adolescents*].’ (HP4, age 26, male)

‘When I ask them [*cases in SRH clinic*], saying “Where do you come from?” They say, “from Gojjam.” “Where do you come from?” They say, “from Wello.” “Where do you come from?” They say, ‘from the South. According to the data of our organisation, the highest level of education recorded is grade 6^th^. They were children who came from the countryside to Addis Ababa in search of job opportunities.’ (HP8, age 40, female)

#### Category 3.2: Gap in law enforcement

This study has shown that there is a gap in law enforcement and a gap in the implementation of legal frameworks that contribute directly or indirectly to risky sexual behaviours. Private businesses and organisations do not adhere to the age limit for using a bar or a hotel. There are also private pharmacies that sell drugs without a prescription, so adolescents get to buy those drugs easily and use them. Adolescents who are younger than 18 years old can get services in hotels and bars and nobody asks them their age. Business organisations do not care about adolescents, rather they care about the money they get. However, some business organisations ask adolescents to show their identification, and adolescents under 18 years old usually show fake identification cards. Even if adolescents’ age is 15, 16 or less, they hire identification (ID) cards from adults by paying money (adults give ID cards as if adolescents are greater than or equal to 18 years old despite their age less than 18 years old). Failure to regulate the sale of drugs from pharmacies makes it easier for adolescents to access them. By using these drugs, adolescents are exposed to risky sexual behaviours. This was derived from the following quotes:

‘Me for example, I am not 18 years old. If I go and ask “give me beer”, they give me. However, on the bottle it says that ‘it could not be sold to a man who is below 18 years old. They do not care if you are 20 years, 18 years or not.’ (FGD2, age 17, male)

‘Making money is their objective. Sometime before, it was said, “It is prohibited for the fewer than 18-year-olds”. But it was adolescents of all ages that would drink. It was simply a talk; otherwise, a business person wouldn’t check their identification cards to serve them beer, will he?’ (HP8, age 40, female)

‘When I go to some places like parties and bars, they ask me for ID. I used to act as if I am 18 years old. I also have a fake ID, I do not know, but my friends brought it [*fake ID*] for me. They told me to pay birrs and I paid that. In some places, they do not even ask for your ID. When they do, we show them our fake IDs.’ (A10, age 17, female)

‘Here are also many pharmacies that sell the drugs without a prescription. For example, my friend has depression and is Tramadol usually prescribed for him. So we share the Tramadol. It cost 20 birrs.’ (A4, age 18, male)

## Discussion

According to the study’s findings, various organisational, community and societal level factors that are perceived as drivers of risky sexual behaviours among adolescents in the urban city of Addis Ababa were explored. The narrative of the study participants revealed resource constraints in the healthcare organisation. In addition to this, both adolescents and health professionals described the gap in knowledge and skill attitude of health professionals serving adolescents. Age differences between adolescents and service providers and fear of confidentiality breaches were stated as barriers to gain knowledge and skills and utilise SRH service. In addition to this poor involvement of the school in SRH, as well as poor controlling and supervision of students in school predispose adolescents to experience romantic sexual relationships and practice in the school compound and school environment as well.

This evidence was supported by different studies. For instance, a study done in Mozambique revealed that the absence of healthcare workers more attuned to the needs of adolescents and young adults was considered a medical barrier to contraceptive use utilisation.^30^ In a study done among adolescents and young people in the urban and rural Democratic Republic of Congo, key barriers to accessing contraception from health facilities and pharmacies included shame, stigma, cost and judgemental attitudes of health providers.^31^

Study participants agreed that they did not get appropriate treatment and care in healthcare institutions and youth centres. A similar study done in Adama, Ethiopia, indicated that pre-college students were not treated according to their level of expectation and that the services provided in the public health institution were not suitable for the youth, rather they perceive the youth centres are designed for adults.^32^ The level of health professional service provided was affected by demographic and socio-cultural factors, because older health professionals were assigned to the adolescent youth-friendly services, Voluntary Counselling and Testing (VCT) services, adolescents and youth centres for adolescents.^33^ Privacy issues hinder adolescents from utilising SRH services^34^ and impact negatively the SRH of young peoples.^35^ Based on a research study done in Harar, East Ethiopia, sex of health professionals, training, judgmental attitude, and lack of up-to-date knowledge of health care providers were identified as factors affecting the utilization of youth-friendly services.^36^

The narratives of the study participants indicated that adolescents start romantic relations and sexual intimacy during their school years. In addition to this, they have easy access to a substance that leads them to risky sexual behaviours. It was also explained that there is a limited level of comprehensive sexuality education about gender, SRH rights, HIV, interpersonal relationships, violence prevention, empowerment, sexual orientation and identity diversity, which enables young people to make informed decisions on healthy, responsible behaviours and mutually protective relationships. A research finding indicated that young people have a right to access accurate, evidence-based, age-appropriate information and education.^37^ However, the lack of comprehensive and tailor-made intervention, poor integration of life skill training, sexuality education and condom programme in school curriculum were listed as some challenges to HIV prevention among adolescent girls and young women in Ethiopia.^38^ In addition to this, it was indicated that there is poor school health education about STIs and HIV and/or AIDS.^39^ Substance abuse was highly prevalent among secondary school students in Ethiopia. Students usually take khat, cigarette and alcohol. Adolescents are able to buy these drugs because they have pocket money and school regulations are not strict, as well as peer pressure.^40^

Study participants revealed that the expansion of provocative business organisations such as night clubs, bars, pensions and traditional, alcohol, hashish and khat houses influences adolescents to experience risky sexual behaviours. Our study is congruent with a study done among preparatory school students in Kolfe-Keranyo sub-city of Addis Ababa which indicated that a quarter of the study participants were substance users. Alcohol, khat and cigarettes were widely used substances. Various factors were associated with substance abuse; believing that substance use improves academic performance, having substance use controlling rules of the school, measures taken by schools for using substances, ineffective (poor) school substance use controlling rules, availability of substance retailing shops around school and pear pressure made them use a substance.^41^ Increased number of hotels, bars, parties, day and night clubs, brothels (houses where men visit prostitutes), pensions and local drink houses are socio-economical drivers that contribute to the high prevalence of HIV epidemic in Addis Ababa.^42^

According to the narrative of the study participants, the community in urban Addis Ababa discourages or does not approve of early sexual intercourse either with one or multiple sexual partners. A study done in Addis Ababa, Ethiopia revealed that closeness to parents and participation in religious activities were associated with lower odds of sexual regret.^43^

But on the other hand, religious conservatism limits sexual activities and restricts SRH access to young people. It is argued that teaching young people information about sex or accessing contraceptives will encourage adolescents to engage in sexual intercourse.^44^ Preparatory and high school students who were not attending religious programs were three point two times more likely to engage in early initiation of sex.^45^ So generally, religious conservatism would be very important to prevent risky sexual behaviours for those who obey the rules of their religion; on the other hand, if adolescents fail to obey the rules of their religion, they will face more problems than other adolescents fail in risky behaviour.

Although there were social taboos about the early initiation of sex, the general community had a negative attitude towards condoms. This type of negative attitude causes fear and shame in buying and carrying condoms among adolescents and finally drives them to have unsafe sex. Based on the study done in Kenya about contraceptive use among adolescents aged between 15 and 19 years, parents and teachers were found to be the barriers to the utilisation of contraceptives.^46^ A negative feeling about carrying a condom predicts lower intention to use a condom, whereas considering a high-risk perception of unsafe sex and higher perceived behaviour control of buying and using a condom predicts better intention of using a condom.^47^

According to this research, adolescents who are jobless and adolescents whose families are poor and urban-rural migrants tend to engage in risky sexual behaviours for material or monetary gain. Families who are unable or who fail to fulfil the basic needs of their children can lead adolescents to have sex for money or material gain.^48^ According to a systematic review done among adolescents in sub-Saharan Africa, those with poor socioeconomic positions were more likely to become pregnant unintentionally because adults exploit this opportunity to establish relationships by meeting the adolescents’ fundamental requirements. This condition creates power imbalances in the negotiation of safe sex resulting in unintended pregnancy during the teenage years and increases the risk of STIs.^49^ Based on research done in Hawassa, Ethiopia, transactional sex and HIV risk among adolescents’ preparatory school girls, more than half of the sexually active respondents, were involved in transactional sex with sugar daddies, and one-fifth of sexually active adolescents had the intention of having transactional sex with sugar daddies. Transaction sex is initiated both by adolescents and sugar daddies. The main reason mentioned for transactional sex was for receiving money or material in the exchange for sex.^50^ Risky sexual behaviours are higher among males who have completed secondary education, have a middle- to high-income wealth index, as well as urban dwellers.^51^ Adolescents and youth who were from poor families practise sex with multiple sexual partners for the sake of incentives, such as money and food.^52^ On the other hand, there are parents who promote sex as a means of getting family income for some young women in households where financial problems prevail.^53^ It was evident that when women are economically dependent, they are forced to migrate from rural to urban areas and engage in risky sexual behaviours.^33^ In addition to this, it was found that premarital sex was higher among school adolescents who come from rural areas than youth living in urban areas in Ethiopia.^54^

Poor law enforcement of age restrictions for adolescents and selling drugs without prescription were listed as the gap in law enforcement that led adolescents to experience risky sexual behaviours indirectly. It was evident that poor implementation of the regulation of alcohol was identified as one of the weaknesses of governmental policies. High consumption of alcohol occurs among young people because of easy access to alcohol. Alcohol is easily accessible because of the poor adherence to laws related to alcohol. So young people engaged in unsafe sex because of high intake of alcohol.^55^

## Conclusion and recommendation

Generally, resource constraints in the healthcare organisation, knowledge skill and attitude gap of health professionals, age difference and fear of confidentiality breach hinder adolescents to utilise and have basic knowledge and skill regarding SRH and access services. In addition to this, poor involvement of the school in SRH, poor control and supervision of students in school predispose adolescents to experience romantic sexual relationships and practice in the school compound and school environment. Poor law enforcement was found especially among private business organisations like bars, hotels, shisha houses, illegal houses and private pharmacies. Adolescents have easy access to substances at any age and this excessive use of substances encourages them to engage in risky sexual behaviours. Neighbourhood and environmental factors, socially related factors and financially related factors are listed at the community level. The living environment of adolescents such as expansion of nightclubs and bars, shish houses and traditional alcohol sellers around the living environment of adolescents, changing the living environment from rural to urban compound or environment led adolescents to risky sexual behaviours. The community has a conservative attitude on early initiation either with single or with multiple sexual partners that also impacts negatively on condom use.

Stakeholders should introduce socially and culturally acceptable comprehensive sexual education curricula for adolescents. Training should be given to health professionals working with adolescents to provide equitable and comprehensive services for adolescents. To overcome social and cultural hurdles affecting teenagers’ sexual behaviour and health, there should be guidelines or techniques on capacity building for families, youth, educators and community members. The regulatory bodies under the health bureau should re-enforce and promote the available laws, policies and regulations that are so as to minimise the exposure of adolescents to risky sexual behaviours for the sake of saving adolescents. Responsible bodies should consider ensuring an adequate and continuous supply of medical equipment, commodities and diagnostic materials to adolescents and youth.
